# Pregnancy-Induced Hypertensive Disorders before and after a National Economic Collapse: A Population Based Cohort Study

**DOI:** 10.1371/journal.pone.0138534

**Published:** 2015-09-17

**Authors:** Védís Helga Eiríksdóttir, Unnur Anna Valdimarsdóttir, Tinna Laufey Ásgeirsdóttir, Arna Hauksdóttir, Sigrún Helga Lund, Ragnheiður Ingibjörg Bjarnadóttir, Sven Cnattingius, Helga Zoëga

**Affiliations:** 1 Centre of Public Health Sciences, Faculty of Medicine, University of Iceland, Reykjavik, Iceland; 2 Department of Epidemiology, Harvard School of Public Health, Boston, Massachusetts, United States of America; 3 Faculty of Economics, University of Iceland, Reykjavik, Iceland; 4 Department of Obstetrics and Gynecology, Landspitali University Hospital, Reykjavik, Iceland; 5 Clinical Epidemiology Unit, Department of Medicine, Solna, Karolinska Institutet, Stockholm, Sweden; University Hospital Basel, SWITZERLAND

## Abstract

**Background:**

Data on the potential influence of macroeconomic recessions on maternal diseases during pregnancy are scarce. We aimed to assess potential change in prevalence of pregnancy-induced hypertensive disorders (preeclampsia and gestational hypertension) during the first years of the major national economic recession in Iceland, which started abruptly in October 2008.

**Methods and Findings:**

Women whose pregnancies resulted in live singleton births in Iceland in 2005–2012 constituted the study population (N = 35,211). Data on pregnancy-induced hypertensive disorders were obtained from the Icelandic Medical Birth Register and use of antihypertensive drugs during pregnancy, including β-blockers and calcium channel blockers, from the Icelandic Medicines Register. With the pre-collapse period as reference, we used logistic regression analysis to assess change in pregnancy-induced hypertensive disorders and use of antihypertensives during the first four years after the economic collapse, adjusting for demographic and pregnancy characteristics, taking aggregate economic indicators into account. Compared with the pre-collapse period, we observed an increased prevalence of gestational hypertension in the first year following the economic collapse (2.4% vs. 3.9%; adjusted odds ratio [aOR] 1.47; 95 percent confidence interval [95%CI] 1.13–1.91) but not in the subsequent years. The association disappeared completely when we adjusted for aggregate unemployment rate (aOR 1.04; 95% CI 0.74–1.47). Similarly, there was an increase in prescription fills of β-blockers in the first year following the collapse (1.9% vs.3.1%; aOR 1.43; 95% CI 1.07–1.90), which disappeared after adjusting for aggregate unemployment rate (aOR 1.05; 95% CI 0.72–1.54). No changes were observed for preeclampsia or use of calcium channel blockers between the pre- and post-collapse periods.

**Conclusions:**

Our data suggest a transient increased risk of gestational hypertension and use of β-blockers among pregnant women in Iceland in the first and most severe year of the national economic recession.

## Introduction

Pregnancy-induced hypertensive disorders, including preeclampsia and gestational hypertension, are significant sources of maternal, foetal and neonatal morbidity [1, 2]. Antenatal psychosocial stress, such as work stress, anxiety and depression, has been found to play a role in the etiology of these hypertensive disorders [3–9]. Another potential source of psychosocial morbidity for pregnant women is a suboptimal socioeconomic situation [10–12], including macroeconomic recessions. A growing body of literature indicates that macroeconomic conditions influence population health [13, 14], including the health of pregnant women [15].

In the autumn 2008, Icelanders experienced a sudden national economic collapse. From being one of the richest nations in the world, the country suffered a major economic breakdown, affecting all inhabitants of Iceland in some way [16]. Recent studies on the health consequences of the Icelandic economic collapse suggest an increase in depressive symptoms [17, 18] and perceived psychological stress [19], as well as an immediate short-term increase in attendance to a cardiac emergency department [20], among women in particular. Furthermore, results from a recent study suggest an increase in low birth weight deliveries during the first year following the Icelandic economic collapse [21]. Low birth weight is more common in pregnancies complicated by hypertensive disorders [22].

We hypothesized that the 2008 economic collapse in Iceland represented a stressor that might have led to a rise in the incidence of pregnancy-induced hypertensive disorders and corresponding drug use among women. Leveraging the nationwide complete registries of all births and drug utilization, we therefore set out to study whether the prevalence of pregnancy-induced hypertensive disorders and use of antihypertensive drugs during pregnancy changed following the major 2008 economic collapse in Iceland. Further, we aimed to assess whether the potential changes in hypertensive disorders and drug use during pregnancy were due to the aggregate economic conditions.

## Methods

### Data sources and study population

The study was based on individual level data from the National Medical Birth Register and Medicines Register in Iceland, linked by personal identification numbers, as well as aggregate data on economic indicators from Statistics Iceland [23]. The Medical Birth Register is a population-based register that covers all births in Iceland since 1972. Registered information includes parental-, pregnancy-, and delivery characteristics as well as birth- and neonatal outcomes [24]. Similarly, the Medicines Register is a nationwide prescription register including information on all prescription drugs dispensed from pharmacies in Iceland since 2003 [25]. Included in the study population were women whose pregnancies had reached at least 20 weeks of gestation on September 27^th^ 2004 and all pregnancies from that time-point, subsequently resulting in live singleton births in Iceland between November 29^th^ 2004 throughout December 31^st^ 2012 (N = 35,211). Pregnancy-induced hypertensive disorders are not diagnosed until after 20 gestational weeks and we therefore limited the cohort entry to this time-point in gestation. Information regarding gestational length were lacking from 20 pregnancies which were excluded from the study population.

### Outcome variables

1. **Pregnancy-induced hypertensive disorders.** Medical conditions during pregnancy are registered in the Medical Birth Register according to the International Classification of Disease, 10^th^ revision [ICD-10][26]. The primary outcomes of the present study were pregnancy-induced hypertensive disorders, including gestational hypertension and preeclampsia. Gestational hypertension [ICD-10 code O13] is defined as newly diagnosed hypertension (systolic blood pressure [SBP] ≥140 mmHg; diastolic blood pressure [DBP] ≥90 mmHg) after 20 weeks of gestation. Preeclampsia is defined as pre-existing or gestational hypertension concurring with significant proteinuria (>300 mg protein in 24 hour urine sample). In the present study, women were classified as having preeclampsia if they had been diagnosed with pre-existing hypertensive disorder with superimposed preeclampsia [ICD-10 code O11], preeclampsia [ICD-10 code O14], or eclampsia [ICD-10 code O15].
2. **Prescription fills of antihypertensive drugs.** As a secondary study outcome we used prescriptions fills for antihypertensive drugs. For the women included in the study, we extracted all prescriptions of β-blockers (Anatomical Therapeutic Chemical (ATC) code C07) and calcium channel blockers (ATC code C08), filled after 20 gestational weeks onwards. Substances belonging to abovementioned ATC drug codes are Labetalol and Nifedipine, which are two of the most commonly used drugs for treating hypertensive disorders during pregnancy [27].

### Explanatory variable

The Icelandic economic collapse was a swift and sudden event, allowing us to pin down the beginning of the crisis to a distinct time point, i.e. the first week of October 2008. In our main analyses, we used calendar time to approximate the economic collapse, categorizing time into a pre-collapse period (unexposed to the economic collapse) and four separate post-collapse years (exposed, to a varying extent, to the economic recession). As pregnancy-induced hypertensive diseases do not occur before 20 weeks of gestation we used ≥20 gestational weeks as an indicator of where to locate each pregnancy with respect to exposure to the economic crisis. The aggregate economic indicators were on quarterly or yearly basis, while the timing of births was on weekly basis (not the actual date of birth). Thus, the beginning of the 4^th^ quarter 2008 (28^th^ of September) was used as a marker of the economic collapse as data on the economic climate showed dramatic changes between the third and fourth quarters of 2008 [23]. The pre-collapse group consisted of pregnancies with a gestational length of 20 weeks or more between September 27^th^ 2004 and September 28^th^ 2008. The four separate post-collapse groups were defined similarly, including all pregnancies having reached at least 20 gestational weeks during i) September 29^th^ 2008–September 27^th^ 2009 (post-collapse year 1), ii) September 28^th^ 2009 –October 3^rd^ 2010 (post-collapse year 2), iii) October 4^th^ 2010 –October 2^nd^ 2011 (post-collapse year 3) and iv) October 3^rd^ 2011 –October 1^st^ 2012 (post-collapse year 4). To further explore the associations between the outcomes of interest (gestational hypertension and β-blockers) and the timing of the economic collapse, we divided the first post-collapse year in two 6 months periods, each including pregnancies with a gestational length of 20 weeks or more during I) September 29^th^ 2008 –March 29^th^ 2009 and II) March 30^th^ 2009 –September 28^th^ 2009.

### Covariates

Information on covariates were retrieved from the Medical Birth Register, including calendar week of birth, gestational length, maternal age at delivery (continuous and grouped by <25; 25–34; ≥35 years), gravidity (primigravida; multigravida), relationship status (cohabitating with other parent; not cohabitating with other parent), maternal place of residence (urban; rural), employment status (employed; student; not working [homemaker/unemployed/on disability]), citizenship (Icelandic; foreign), diabetes mellitus (pre-existing diabetes [ICD-10 code O24.0–24.3]; gestational diabetes [ICD-10 code O24.4–24.9]), pre-existing hypertension [ICD-10 code O10] and infant’s sex [28]. Gestational length was determined by ultrasound measurement before the 21^st^ week of gestation for 99.9% of the study population. For 37 pregnancies, gestational length was based on women’s self-reported last menstrual period. In order to control for seasonality, we used quarters of the calendar year.

In order to further capture if the potential influence of time on the outcomes was due to macroeconomic changes, we used aggregate economic indicators as the unit of analysis. The economic indicators tested were national unemployment rate and gross domestic product (GDP), as traditional measures of economic conditions. However, as the Icelandic collapse resulted in a debt crisis at the national and individual levels [16], we also tested the balance of accounts and rates of homes that finds it very difficult to make ends meet (i.e. to cover expenses through the pay period) or have defaults on loans or rent. The unemployment rate and GDP were on a quarterly basis while the other indicators were on a yearly basis.

### Data analysis

We calculated descriptive statistics for all maternal and pregnancy-related characteristics, contrasting frequencies across exposure groups. Chi-square and one-way ANOVA were used to assess differences in the distribution of maternal and pregnancy-related characteristics.

In order to examine the impact of the economic collapse on the outcome variables, we used logistic regression analyses to calculate the crude and adjusted odds ratios (ORs) and corresponding 95% confidence intervals [CIs] for each outcome contrasting the four separate post-collapse years with the pre-collapse period. The total number of calendar weeks during the study period, beginning on September 27^th^ 2004, was used to address potential time-trends in the aggregate explanatory variables as well as in the prevalence of the outcome variables. The final regression models were adjusted for I) maternal age, gravidity and time-trend, II) maternal age, gravidity, time-trend, diabetes, pre-existing hypertension, relationship status, place of residence, employment status, citizenship and infant sex, III) maternal age, gravidity, time-trend and aggregate economic indicators, if associated with index outcome in a supplementary analysis. We did not adjust for seasonality in our main regression models as the exposure categories were equally balanced with respect to calendar time. However, the effect of seasonality on the outcomes of interest was explored in a supplementary analysis. Similarly, adjusting for seasonality, logistic regression models were created in order to further explore the odds ratio of gestational hypertension and prescription fills for β-blockers in months 1–6 and months 7–12 following the economic collapse, compared with the pre-collapse period.

Further, in a supplementary analysis we used logistic regression analyses to estimate the crude and adjusted ORs and corresponding 95% CIs to assess the association between each aggregate economic indicator, i.e. unemployment rate, GDP, balance of accounts, rates of either finding it very difficult making ends meet or having defaults on loans or rent, and the study outcomes irrespective of the defined exposure categories. We adjusted for all maternal and pregnancy-related covariates separately to determine which of the covariates affected the measured association. We then constructed three different regression models adjusting for i) time-trend, ii) time-trend, maternal age and gravidity and iii) time-trend, maternal age, gravidity, diabetes, pre-existing hypertension [ICD-10 code O10], relationship status, place of residence, employment status, citizenship and infant sex.

All the data analyses were performed using IBM SPSS Statistic 20.

The study was approved by the Icelandic National Bioethics committee (VSNb2013010002/03.07), the Data Protection Authority (2012121499HGK/—) and the Directorate of Health (1301064/5.6.1/gkg). An informed consent from women in the study population was not obtained as all personal information was anonymized and de-identified prior to analysis.

## Results

Table 1 shows the demographic and pregnancy-related characteristics of women across exposure categories. After the economic collapse, pregnant women in Iceland were slightly older, less likely to be employed, less likely to be of Icelandic citizenship and less likely to cohabit with the other parent, as compared with pregnant women before the collapse (Table 1).

**Table 1:** Maternal background characteristics of live singleton births in the study population in Iceland between November 29^th^ 2004 and December 31^st^ 2012.

Maternal & obstetric	Pre-collapse years^a^		Post-collapse year 1^b^		Post-collapse year 2^c^		Post-collapse year 3^d^		Post-collapse year 4^e^		*p*-value
characteristics	N = 17,447		N = 4,990		N = 4,664		N = 4,295		N = 3,815		
*Mean maternal age [SD]*	28.92 [5.50]		29.12 [5.45]		29.40 [5.47]		29.27 [5.45]		29.45 [5.43]		<0.001*
	**Births, *n***	**Births, %**	**Births, *n***	**Births, %**	**Births, *n***	**Births, %**	**Births, *n***	**Births, %**	**Births, *n***	**Births, %**	
*Maternal age (years)*											
<25	3,915	22.4	1,021	20.5	904	19.4	855	19.9	733	19.2	<0.001**
25–34	10,600	60.8	3,111	62.3	2,861	61.3	2,664	62.0	2,364	62.0	
≥35	2,932	16.8	858	17.2	899	19.3	776	18.1	718	18.8	
*Gravidity*											
Primigravida	7,075	40.6	2,021	40.5	1,840	39.5	1,662	38.7	1,523	39.9	0.184
Multigravida	10,372	59.4	2,969	59.5	2,824	60.5	2,633	61.3	2,292	60.1	
*Relationship status*											
Cohabitating with other parent	14,872	85.2	4,192	84.0	3,893	83.5	3,478	81.0	3,080	80.7	0.006
Not cohabitating with other parent	2,442	14.0	744	14.9	721	15.5	645	15.0	575	15.1	
Missing	133	0.8	54	1.1	50	1.1	172	4.0	160	4.2	
*Maternal place of residence*											
Capital area	11,244	64.4	3,328	66.7	3,054	65.5	2,810	65.4	2,489	65.2	0.510
Rural area	5,936	34.0	1,653	33.1	1,594	34.2	1,464	34.1	1,311	34.4	
Missing	267	1.5	9	0.2	16	0.3	21	0.5	15	0.4	
*Maternal employment status*											
Employed	13,123	75.2	3,665	73.4	3,312	71.0	3,027	70.5	2,689	70.5	<0.001
Student	2,841	16.3	836	16.8	834	17.9	758	17.6	717	18.8	
Homemaker/on disability/unemployed	1,438	8.2	489	9.8	518	11.1	509	11.9	406	10.6	
Missing	45	0.3	0	0	0	0	1	<0.001	3	0.1	
*Maternal citizenship*											
Icelandic	15,886	91.1	4,332	86.8	4,105	88.0	3,769	87.8	3,336	87.4	<0.001
Foreign	1,561	8.9	658	13.2	559	12.0	526	12.2	479	12.6	
*Infant‘s sex*											
Boy	8,975	51.4	2,531	50.7	2,423	52.0	2,258	52.6	1,954	51.2	0.447
Girl	8,467	48.5	2,459	49.3	2,240	48.0	2,037	47.4	1,861	48.8	
Missing	5	<0.001	0		1	<0.001	0		0		
*Diabetes*											
Pre-existing diabetes	63	0.4	22	0.4	19	0.4	17	0.4	21	0.6	0.593
Gestational diabetes	485	2.8	196	3.9	188	4.0	195	4.5	161	4.2	<0.001
*Hypertensive disorders of pregnancy*											
Pre-existing hypertension	212	1.2	69	1.4	56	1.2	47	1.1	46	1.2	<0.489
Pregnancy-induced hypertension***	1,003	5.7	318	6.4	270	5.8	261	6.1	228	6.0	0.540
Gestational hypertension	422	2.4	193	3.9	144	3.1	133	3.1	117	3.1	<0.001
Preeclampsia	629	3.6	141	2.8	141	3.0	138	3.2	126	3.3	0.048
*Cardiovascular drugs*											
β-blockers (C07)	336	1.9	156	3.1	115	2.5	92	2.1	106	2.8	<0.001
Calcium channel blockers (C08)	71	0.4	34	0.7	28	0.6	40	0.9	60	1.6	<0.001

*Based on one-way ANOVA.

**Based on chi-square test.

*** Of those diagnosed with pregnancy-induced hypertensive disorder 48; 16; 15; 10; 15 were both diagnosed with gestational hypertension and preeclampsia

Included in the collapse groups are women with singleton pregnancies with gestational length of 20 weeks or more during ^a^September 27^th^ 2004–September 28^th^ 2008, ^b^September 29^th^ 2008 –September 27^th^ 2009, ^c^September 28^th^ 2009 –October 3^rd^ 2010, ^d^October 4^th^ 2010 –October 2^nd^ 2011, ^e^October 3^rd^ 2011 –October 1^st^ 2012.

**Fig 1:**
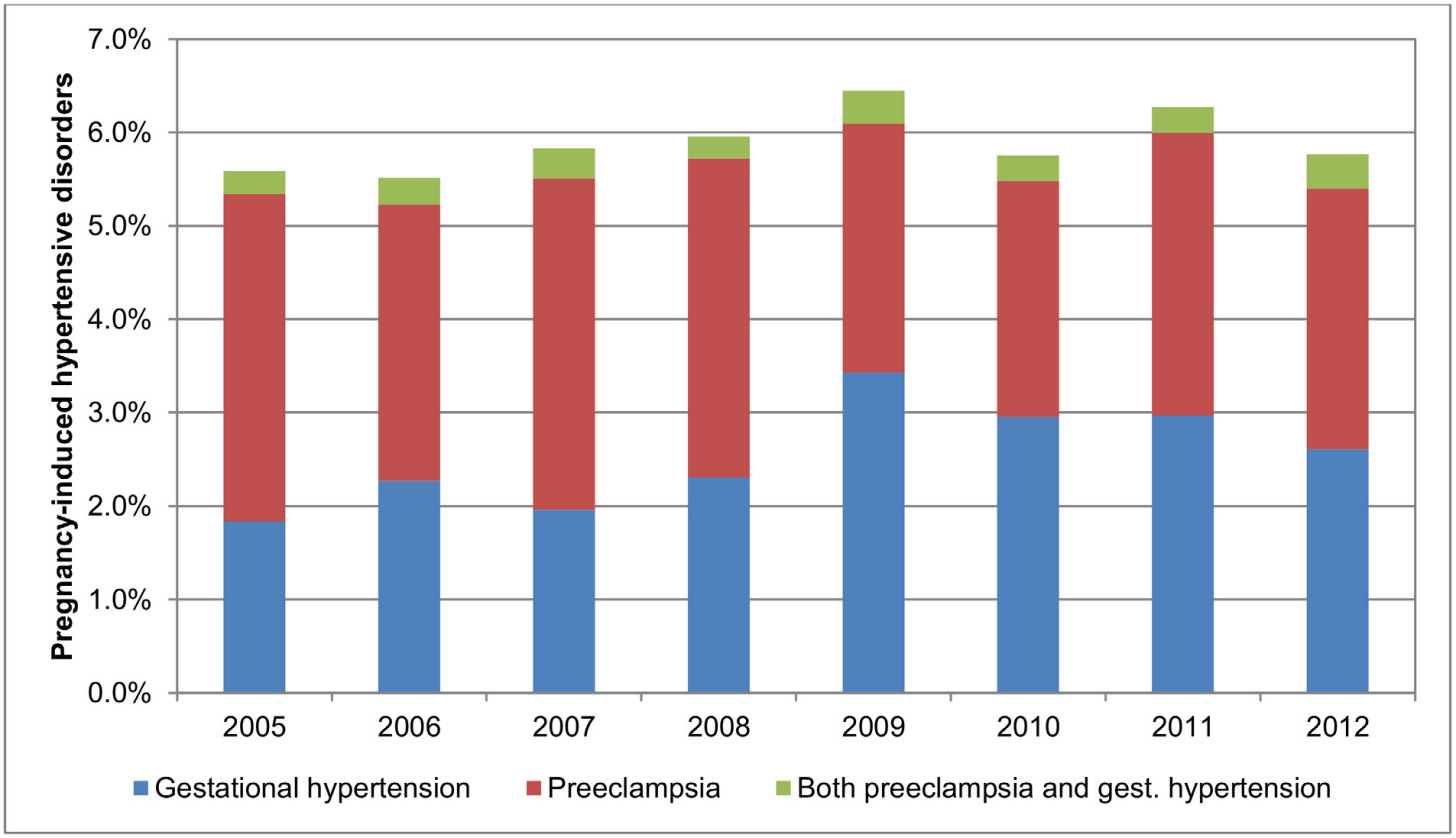
Prevalence of overall pregnancy-induced hypertensive disorders, gestational hypertension and preeclampsia in Iceland, 2005–2012.

**Fig 2:**
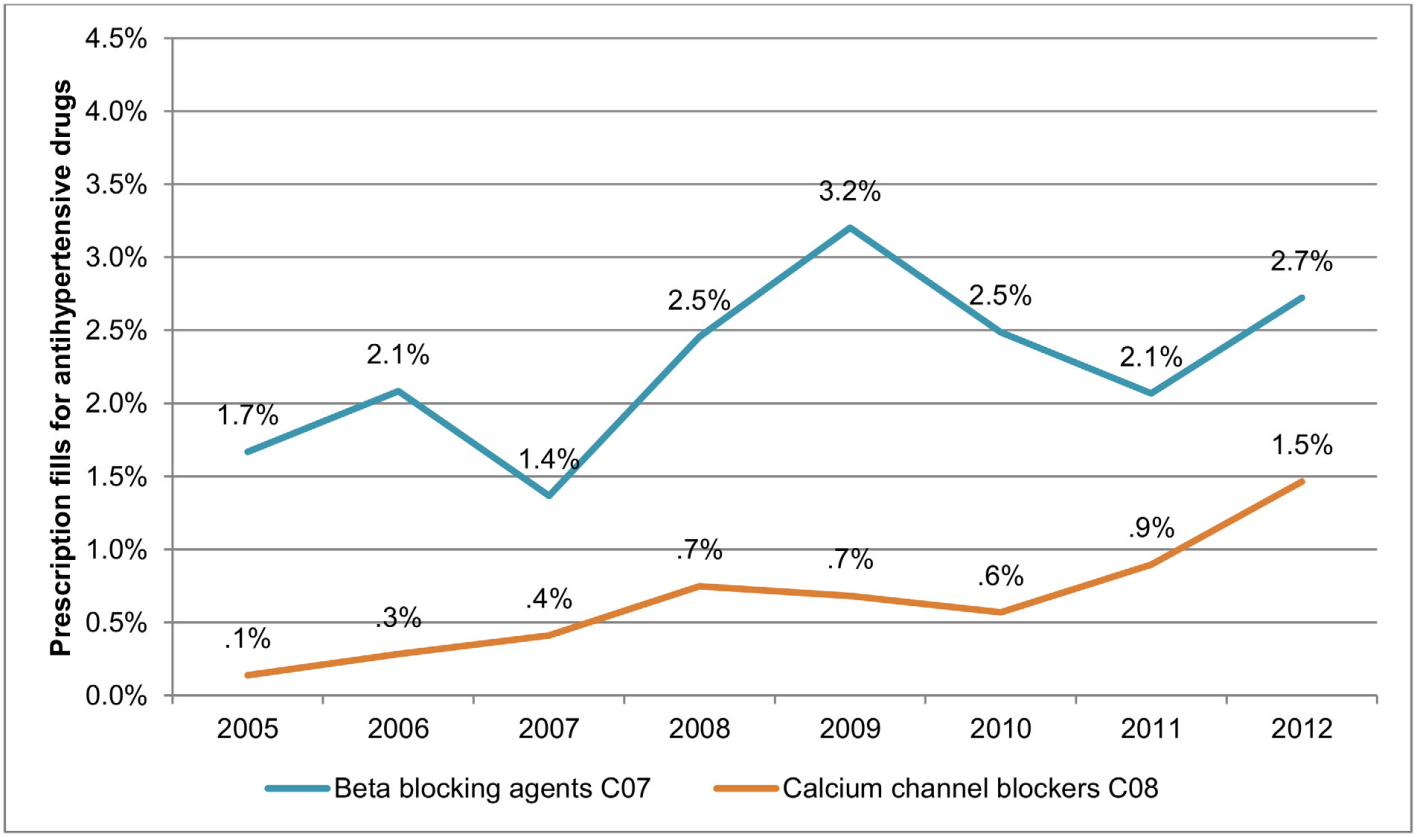
Prevalence of prescription fills for β-blockers and calcium channel blockers among pregnant women in Iceland 2005–2012, after gestational week 20.

The overall prevalence of pregnancy-induced hypertensive disorders remained stable during the study period. However, when gestational hypertension and preeclampsia were examined separately, an increase was observed for gestational hypertension from 2.1% (74 of 4,166) to 2.6% (114 of 4,383), while the prevalence of preeclampsia decreased from 3.8% (159 of 4,166) to 3.2% (139 of 4,383) (Fig 1). During the whole study period, 0.3% (104/35,211) of women were diagnosed with both gestational hypertension and preeclampsia, a proportion which was equal across exposure categories (Table 1). Fig 2 shows an increase over time in the prevalence of filled prescriptions for antihypertensive drugs among the study population.

After adjusting for time-trend and other available covariates, we observed increased odds of pregnancy-induced hypertensive disorders (aOR = 1.03 [1.00–1.06]), gestational hypertension (aOR = 1.09, 95% CI [1.05–1.13]) and prescription fills for β-blockers (aOR = 1.08, 95% CI [1.04–1.13]) with each percentage point increase in unemployment (S1 Appendix). Unemployment rate was not associated with preeclampsia or prescription fills for calcium channel blockers. No associations were observed between the other aggregate economic indicators and outcomes of interest (S1 Appendix).

Adjusting for maternal age, gravidity and time-trend, we observed a statistically significant increase in gestational hypertension during post-collapse year 1 (aOR_year 1_ = 1.47, 95% CI [1.13–1.91]), as compared with the pre-collapse period (Table 2). Adding other maternal- and pregnancy related covariates did not affect this association, while it disappeared (aOR_year1_ = 1.04, 95% CI [0.74–1.47]) when adding aggregate unemployment rate to the model. We found no association between the economic collapse and gestational hypertension during the subsequent post-collapse years or with overall pregnancy-induced hypertensive disorders or preeclampsia during any of the post-collapse years (Table 2).

**Table 2:** The odds ratios [OR] and 95% confidence intervals [CI] of (A) overall pregnancy-induced hypertensive disorders, (B) gestational hypertension and (C) preeclampsia in each of the four post-collapse years following the economic collapse in Iceland compared with pre-collapse period.

Regression models	Pre-collapse period^a^	Post-collapse year 1^b^	Post-collapse year 2^c^	Post-collapse year 3^d^	Post-collapse year 4^e^
	OR [95% CI]	OR [95% CI]	OR [95% CI]	OR [95% CI]	OR [95% CI]
***(A) Pregnancy-induced hypertensive disorders***					
Crude	1.00 [ref.]	1.12 [0.98–1.27]	1.01 [0.88–1.16]	1.06 [0.92–1.22]	1.04 [0.90–1.21]
Model I*	1.00 [ref.]	1.13 [0.94–1.37]	1.03 [0.82–1.30]	1.11 [0.84–1.47]	1.08 [0.78–1.50]
Model II**	1.00 [ref.]	1.13 [0.93–1.36]	1.02 [0.81–1.29]	1.10 [0.83–1.46]	1.06 [0.76–1.48]
Model III***	1.00 [ref.]	0.96 [0.74–1.23]	0.86 [0.65–1.15]	0.96 [0.70–1.31]	0.99 [0.70–1.38]
***(B) Gestational hypertension***					
Crude	1.00 [ref.]	1.62 [1.37–1.93]	1.29 [1.06–1.56]	1.29 [1.06–1.57]	1.28 [1.04–1.57]
Model I*	1.00 [ref.]	1.47 [1.13–1.91]	1.11 [0.79–1.55]	1.09 [0.72–1.63]	1.02 [0.63–1.64]
Model II**	1.00 [ref.]	1.44 [1.11–1.87]	1.08 [0.77–1.52]	1.05 [0.69–1.58]	0.99 [0.61–1.61]
Model III***	1.00 [ref.]	1.04 [0.74–1.47]	0.78 [0.52–1.17]	0.81 [0.52–1.26]	0.85 [0.52–1.38]
***(C) Preeclampsia***					
Crude	1.00 [ref.]	0.78 [0.65–0.94]	0.83 [0.69–1.00]	0.89 [0.74–1.07]	0.91 [0.75–1.11]
Model I*	1.00 [ref.]	0.86 [0.67–1.11]	0.97 [0.72–1.31]	1.09 [0.76–1.56]	1.15 [0.76–1.74]
Model II**	1.00 [ref.]	0.86 [0.66–1.10]	0.97 [0.71–1.31]	1.11 [0.77–1.59]	1.12 [0.73–1.72]
Model III***	1.00 [ref.]	0.88 [0.62–1.23]	0.99 [0.67–1.45]	1.11 [0.73–1.67]	1.16 [0.75–1.79]

* Adjusted for maternal age, gravidity and time in weeks [time-trend].

** Simultaneously adjusted for maternal age, gravidity, time in weeks, sex, diabetes, pre-existing hypertension, relationship status, place of residence, employment status and citizenship.

***Adjusted for maternal age, gravidity, time in weeks and aggregate unemployment rate.

Included in the collapse groups are women with singleton pregnancies with gestational length of 20 weeks or more during ^a^September 27^th^ 2004–September 28^th^ 2008, ^b^September 29^th^ 2008 –September 27^th^ 2009, ^c^September 28^th^ 2009 –October 3^rd^ 2010, ^d^October 4^th^ 2010 –October 2^nd^ 2011, ^e^October 3^rd^ 2011 –October 1^st^ 2012.

The pattern of prescription fills of β-blockers across exposure categories was similar to what was observed for gestational hypertension (Table 3). The odds of filling a prescription for β-blockers were increased by 43% only during post-collapse year 1 (aOR_year 1_ = 1.43, 95% CI [1.07–1.90]); an increase which disappeared when adjusting for aggregate unemployment rate (Table 3).

**Table 3:** The odds ratios [OR] and 95% confidence intervals [CI] of (A) prescription fills for β-blockers and (B) prescription fills for calcium channel blockers in pregnancies during each of the four post-collapse years following the economic collapse in Iceland compared with pre-collapse period.

Regression models	Pre-collapse period^a^	Post-collapse year 1^b^	Post-collapse year 2^c^	Post-collapse year 3^d^	Post-collapse year 4^e^
	OR [95% CI]	OR [95% CI]	OR [95% CI]	OR [95% CI]	OR [95% CI]
***(A) β-blockers (C07)***					
Crude	1.00 [ref.]	1.64 [1.36–1.99]	1.29 [1.04–1.60]	1.12 [0.88–1.41]	1.46 [1.17–1.82]
Model I*	1.00 [ref.]	1.43 [1.07–1.90]	1.05 [0.72–1.52]	0.87 [0.55–1.37]	1.06 [0.62–1.80]
Model II**	1.00 [ref.]	1.49 [1.11–2.00]	1.12 [0.76–1.64]	0.91 [0.56–1.46]	1.10 [0.64–1.89]
Model III***	1.00 [ref]	1.05 [0.72–1.54]	0.77 [0.49–1.20]	0.67 [0.41–1.10]	0.91 [0.53–1.55]
***(B) Calcium channel blockers***					
Crude	1.00 [ref.]	1.68 [1.11–2.53]	1.48 [0.95–2.29]	2.30 [1.56–3.39]	3.91 [2.77–5.52]
Model I*	1.00 [ref.]	0.67 [0.38–1.18]	0.38 [0.18–0.81]	0.40 [0.16–0.97]	0.45 [0.16–1.31]
Model II**	1.00 [ref.]	0.62 [0.35–1.11]	0.36 [0.17–0.76]	0.32 [0.13–0.79]	0.39 [0.13–1.14]
Model III***	1.00 [ref.]	1.07 [0.49–2.31]	0.63 [0.25–1.62]	0.60 [0.21–1.67]	0.59 [0.19–1.81]

* Adjusted for maternal age, gravidity and time in weeks [time-trend].

** Simultaneously adjusted for maternal age, gravidity, time in weeks, sex, diabetes, pre-existing hypertension, relationship status, place of residence, employment status and citizenship.

***Adjusted for maternal age, gravidity, time in weeks and aggregate unemployment rate.

Included in the collapse groups are women with singleton pregnancies with gestational length of 20 weeks or more during ^a^September 27^th^ 2004–September 28^th^ 2008, ^b^September 29^th^ 2008 –September 27^th^ 2009, ^c^September 28^th^ 2009 –October 3^rd^ 2010, ^d^October 4^th^ 2010 –October 2^nd^ 2011, ^e^October 3^rd^ 2011 –October 1^st^ 2012

Furthermore, the economic collapse did not seem to impact on the prevalence of prescription fills for calcium channel blockers beyond the observed time trend of increase; after adjustment for maternal age, gravidity and time-trend, the positive crude increase in calcium channel blockers disappeared and even reversed in post-collapse year 2 (Table 2). Further adjustment for seasonality did not significantly change the association between the economic collapse and the outcomes of interest (S2 Appendix and S3 Appendix). When exploring the odds ratios of gestational hypertension and prescription fills for β-blockers in finer time-period during the first year following the economic collapse, we observed similar point estimates for months 1–6 and months 7–12 (gestational hypertension: aOR_months1–6_ = 1.36, 95% CI [1.00–1.85]; aOR_months7–12_ = 1.44, 95% CI [1.05–1.97] and β-blockers: aOR_months1–6_ = 1.37, 95% CI [0.98–1.92]; aOR_months7–12_ = 1.31, 95% CI [0.92–1.85]) (S4 Appendix).

## Discussion

In this nationwide study of women with live singleton births in Iceland during a time of severe economic fluctuation, we observed a transient increase in the risk of gestational hypertension and prescription fills for β-blockers during the first year following the economic collapse, but no such increase was detected for preeclampsia. In parallel with the development of the macroeconomic conditions, this initial increase in gestational hypertension seemed to level off with years passing from the severe economic collapse. Indeed, our findings suggest that the increase risk of gestational hypertension at the first year of the crisis may be explained by changing aggregate unemployment rates, reflecting the severity of the economic crisis at this time point.

The dramatic economic collapse in Iceland in 2008 affected the whole Icelandic population in one way or another and recent studies suggest considerable influence on mental health [17–19] and reproductive outcomes [21]. Our findings on increased risk of gestational hypertension and corresponding drug use after the economic collapse add to this litterature and are in line with the findings of previous studies indicating an association between psychosocial stress and pregnancy-induced hypertensive disorders [3–9]. Most [3–9], but not all studies [29], have found high levels of perceived stress, work-related stress, depression and anxiety to be associated with pregnancy-induced hypertensive disorders. A large population based study by László et al. found severe psychosocial stress to be associated with a modest increase in preeclampsia [7], while other hypertensive diseases of pregnancy were not reported. We did not observe any association between the economic collapse and risk of preeclampsia.

The pathways through which stress could affect pregnancy-induced hypertensive disorders are not fully understood. Stress can influence health behaviours of affected individuals to the worse, resulting in higher levels of smoking, less exercise and poorer diet [30]. In addition, physiology of individuals experiencing stress may also be affected [12]. Over-activation of the hypothalamic-pituitary-adrenal axis and the autonomous nervous system at times of stress leads to a corresponding rise in the concentrations of cortisol and nor-epinephrine, which act upon different organ systems and may cause pathophysiological changes, including hypertension and cardiovascular disease in non-pregnant populations [12]. Such pathophysiologic reactions, in combination with altered behaviours of pregnant women, may offer explanation to our findings.

It is unclear why we observed an association between the economic collapse and gestational hypertension but not to preeclampsia, yet two explanations seem most plausible. Firstly, if viewed as separate diseases with different aetiologies, it is possible that mechanisms underlying gestational hypertension are more sensitive to stress compared with mechanisms underlying preeclampsia. Secondly, from the perspective that the gestational hypertension is merely a milder form of preeclampsia, it is possible that the economic collapse was not a stressor of sufficient magnitude to cause the more severe form of the disorder.

The β-blocking agent Labetalol is the first line of treatment of pregnancy-induced hypertensive disorders in Iceland. In most cases the calcium channel blocker, Nifedipine, is prescribed if treatment with Labetalol has proven insufficient or is not tolerated by the pregnant woman. However, during the last 10 years, Nifedipine has increasingly been used for treatment of contractions during pregnancy, which might explain the time-trend observed for calcium channel blockers in our study.

The available data in our study are not informative on potential underlying mechanisms although they suggest that the influence of this societal stressor on pregnancy-induced hypertensive disorders was rapid and transient, although further exploitation of the time during the first year following the collapse did not reveal much difference of the effect between women who were pregnant in the first and second half-year after the collapse. The observed increase in gestational hypertension and in prescription fills for β-blockers in the first year following the economic collapse disappeared entirely when we adjusted for the aggregate unemployment level. This finding is supported by a line of studies linking pregnancy-induced hypertensive disorders, particularly preeclampsia, to socioeconomic difficulties [31–35]. To our knowledge, our study is the first to examine the effect of a major macroeconomic downturn on pregnancy-induced hypertensive disorders. Yet, there are some studies [14, 36–38], although not all [39–41], that have provided evidence of increased cardiovascular morbidity and mortality in the general population during times when the economy is bad. Based on self-reports from a nationally representative cohort of approximately 4000 Icelanders from the Health and Well-being study, Asgeirsdottir et al. reported a modest increase in self-reported hypertension in the first year following the economic collapse in 2008 among men, but not among women [42]. However, it is indeed possible that pregnant women are particularly vulnerable to the socioeconomic adversities brought about by a national economic crisis.

### Study strengths and limitations

This study leverages the National Medical Birth Register and Medicines Register in Iceland to accomplish a population based cohort study of all pregnancies resulting in live singleton births in Iceland during eight years of follow-up. These registries are a rich source of information regarding health and drug use of pregnant women and their offspring, enabling collection of data independent of the exposure categories, i.e. timing of the economic collapse. The detailed information in the Medical Birth Register allowed us to account for potential confounding factors. In order to enhance internal validity of the study, we restricted the study population to singleton pregnancies as previous studies indicate higher risk of hypertensive disorders in multiple pregnancies. Furthermore, we adjusted for potential time-trend in the study outcomes in the statistical analyses. Thus, the observed increase in gestational hypertension and prescription fills for β-blockers is unlikely to be explained by secular trends in the detection rate of the diseases or prescribing practices among health care professionals in Iceland.

A limitation of this study is the lack of accessible data on smoking, body mass index and other behavioral factors that might possibly influence the observed increase in gestational hypertension. Many of these behavioral risk factors are strongly associated with pregnancy-induced hypertensive disorders, although some in opposite direction [43]. A sudden decrease in maternal smoking or increase in overweight or obesity among women of childbearing age, could theoretically have contributed to the increase in the prevalence of gestational hypertension among women who were pregnant in the first year following the economic collapse. However, in a recent study on the prevalence of smoking, overweight and obesity among pregnant women in Iceland between 2001 and 2010, a reduction in maternal smoking was observed whereas the mean body mass index of pregnant women remained relatively stable [44]. The same decreasing trend in smoking has been reported among the general population following the economic collapse in Iceland [45, 46]. Loss of resources may lead to worsened nutrition or decreased attention to personal health [47]. In recent studies of health behaviors of Icelanders following the economic collapse, a decrease in health-compromising behaviors, such as smoking, consumption of fast food, sugar-sweetened beverages, sweets or alcohol, was reported. Some health-promoting factors were found to increase after the collapse, such as consumption of fish oil and getting enough sleep, while the intake of fruits and vegetables decreased [45, 48]. Thus, we cannot rule out that a sudden nutritional deterioration or an altered risk behavior may have contributed to the observed increase in gestational hypertension. A second limitation pertains to the fact that only pregnancies resulting in live births were included in the study. Women with hypertensive disorders of pregnancy have higher rates of stillbirths than women with normal blood pressure [49]. Therefore, the observed increase in gestational hypertension in the first year following the economic collapse might have led to an increase in stillbirths, which potentially could have resulted in an underestimation of the observed effect. However, the stillbirth rate in Iceland is very low, on average 3.5 per 1000 births during the study period [24]. A rate of this magnitude is unlikely to significantly impact the observed effect estimates. Thirdly, we cannot rule out potential misclassification of the hypertensive outcomes. It is possible that women with preeclampsia were temporarily misclassified as having gestational hypertension, explaining the observed increase in the first year following the economic collapse. However, such misclassification is unlikely as preeclampsia is a severe condition that poses both mother and fetus at high risk, requiring high intensity maternal care. Moreover, it is unlikely that such misclassification would be limited only to the first year following the collapse. Fourthly, as this is a quasi-experimental design we are unable to control for other potential factors that might have been occurring at the same time as the economic collapse, affecting the outcomes of interest. However, the collapse of the financial system in Iceland was by far the most notable event occurring at that time and other important changes that may have occurred in Iceland around that time are likely to be consequences of the collapse. Lastly, our study is based on an entire nation exposed to a specific economic collapse. All economic fluctuations have their own characteristics, the distinct features of the collapse in Iceland included currency- and household debt crisis. Thus, these findings cannot be readily generalized to other populations of pregnant women undergoing economic recessions.

## Conclusions

In summary, the results suggest a transient increase in gestational hypertension and use of β-blockers among pregnant women in Iceland in the first year following the national economic collapse. The severity of the aggregate economic climate at that time with a gradual yet slow recovery during the following years is a likely explanation for the observed pattern.

## Supporting Information

S1 Appendix The association between aggregate macroeconomic indicators in Iceland and (A) overall pregnancy-induced hypertensive disorders, (B) gestational hypertension, (C) preeclampsia, (D) prescription fills for β-blockers, (E) prescription fills for calcium channel blockers, among the women in the study population giving birth to live born singletons between November 29^th^ 2004 and December 31^st^ 2012.
(DOCX)

S2 Appendix The odds ratios [OR] and 95% confidence intervals [CI] of (A) overall pregnancy-induced hypertensive disorders, (B) gestational hypertension and (C) preeclampsia in each of the four post-collapse years following the economic collapse in Iceland compared with pre-collapse period, adjusted for seasonality.
(DOCX)

S3 Appendix The odds ratios [OR] and 95% confidence intervals [CI] of (A) prescription fills for β-blockers and (B) prescription fills for calcium channel blockers in pregnancies during each of the four post-collapse years following the economic collapse in Iceland compared with pre-collapse period, adjusted for seasonality.
(DOCX)

S4 Appendix The odds ratios [OR] and 95% confidence intervals [CI] of (A) gestational hypertension and (B) β-blockers in the first and second half year following the economic collapse in Iceland compared with pre-collapse period
(DOCX)
