# Supplementary material for: Pregnancy-Induced Hypertensive Disorders before and after a National Economic Collapse: A Population Based Cohort Study
Source: PLoS One. 2015 Sep 17;10(9):e0138534. doi: 10.1371/journal.pone.0138534 (PMC4575018; doi:10.1371/journal.pone.0138534)
Supplement: S1 Appendix — The association between aggregate macroeconomic indicators in Iceland and (A) overall pregnancy-induced hypertensive disorders, (B) gestational hypertension, (C) preeclampsia, (D) prescription fills for β-blockers, (E) prescription fills for calcium channel blockers, among the women in the study population giving birth to live born singletons between November 29th 2004 and December 31st 2012. (DOCX) [file pone.0138534.s001.docx]

**S1 Appendix.** The association between aggregate macroeconomic indicators in Iceland and (A) overall pregnancy-induced hypertensive disorders, (B) gestational hypertension, (C) preeclampsia, (D) prescription fills for β-blockers, (E) prescription fills for calcium channel blockers, among the women in the study population giving birth to live born singletons between November 29^th^ 2004 and December 31^st^ 2012

|  | **Regression models** | | | |
| --- | --- | --- | --- | --- |
|  | **Crude** | **Model I*** | **Model II**** | **Model III***** |
|  | **OR [95% CI]** | **OR [95% CI]** | **OR [95% CI]** | **OR [95% CI]** |
| ***(A) Pregnancy-induced hypertensive disorders*** |  |  |  |  |
| Aggregate unemployment (%)^α^ | 1.018 [0.999-1.037] | 1.025 [0.997-1.053] | 1.029 [1.001-1.057] | 1.029 [1.001-1.058] |
| Gross domestic product (milliard ISK) ^α^ | 1.000 [0.999-1.001] | 1.000 [0.997-1.002] | 0.999 [0.996-1.002] | 0.999 [0.996-1.002] |
| Balance of account (milliard ISK) ^β^ | 1.000 [1.000-1.001] | 1.000 [0.999-1.001] | 1.000 [0.999-1.001] | 1.000 [0.999-1.001] |
| Very difficult making ends meet (%)^β^ | 1.004 [0.990-1.018] | 0.997 [0.974-1.021] | 1.000 [0.977-1.024] | 0.999 [0.987-1.012] |
| Defaults on loans or rent (%)^β^ | 1.003 [0.993-1.013] | 1.000 [0.985-1.016] | 1.002 [0.987-1.018] | 1.003 [0.987-1.018] |
| ***(A) Gestational hypertension*** |  |  |  |  |
| Aggregate unemployment (%)^α^ | 1.082 [1.053-1.111] | 1.085 [1.045-1.126] | 1.089 [1.049-1.130] | 1.088 [1.047-1.129] |
| Gross domestic product (milliard ISK) ^α^ | 1.002 [1.001-1.003] | 0.999 [0.996-1.003] | 0.999 [0.995-1.003] | 0.999 [0.995-1.003] |
| Balance of account (milliard ISK) ^β^ | 1.001 [1.000-1.002] | 1.000 [0.999-1.001] | 1.000 [0.999-1.001] | 1.000 [0.999-1.001] |
| Very difficult making ends meet (%)^β^ | 1.036 [1.016-1.056] | 1.012 [0.979-1.046] | 1.014 [0.981-1.048] | 1.012 [0.979-1.046] |
| Defaults on loans or rent (%)^β^ | 1.026 [1.012-1.041] | 1.010 [0.989-1.032] | 1.012 [0.990-1.034] | 1.011 [0.989-1.033] |
| ***(B) Preeclampsia*** |  |  |  |  |
| Aggregate unemployment (%)^α^ | 0.964 [0.941-0.989] | 0.969 [0.934-1.005] | 0.972 [0.937-1.009] | 0.973 [0.937-1.010] |
| Gross domestic product (milliard ISK) ^α^ | 0.999 [0.998-1.000] | 0.999 [0.996-1.003] | 0.999 [0.995-1.002] | 0.999 [0.995-1.002] |
| Balance of account (milliard ISK) ^β^ | 0.999 [0.999-1.000] | 1.000 [0.999-1.001] | 1.000 [0.999-1.001] | 1.000 [0.999-1.001] |
| Very difficult making ends meet (%)^β^ | 0.976 [0.958-0.994] | 0.982 [0.951-1.013] | 0.985 [0.955-1.017] | 0.987 [0.956-1.019] |
| Defaults on loans or rent (%)^β^ | 0.984 [0.971-0.997] | 0.991 [0.972-1.011] | 0.993 [0.974-1.013] | 0.994 [0.975-1.015] |
| ***C) β-blockers*** |  |  |  |  |
| Aggregate unemployment (%)^α^ | 1.077 [1.045-1.109] | 1.070 [1.026-1.115] | 1.072 [1.028-1.118] | 1.083 [1.038-1.131] |
| Gross domestic product (milliard ISK) ^α^ | 1.003 [1.001-1.004] | 1.002 [0.998-1.006] | 1.002 [0.998-1.006] | 1.002 [0.998-1.006] |
| Balance of account (milliard ISK) ^β^ | 1.000 [0.999-1.001] | 0.999 [0.998-1.000] | 0.999 [0.998-1.000] | 0.999 [0.998-1.000] |
| Very difficult making ends meet (%)^β^ | 1.025 [1.003-1.048] | 0.977 [0.941-1.014] | 0.978 [0.942-1.016] | 0.985 [0.948-1.024] |
| Defaults on loans or rent (%)^β^ | 1.015 [0.999-1.031] | 0.982 [0.958-1.006] | 0.983 [0.959-1.007] | 0.988 [0.963-1.013] |
| ***D) Calcium channel blockers*** |  |  |  |  |
| Aggregate unemployment (%)^α^ | 1.092 [1.034-1.153] | 0.892 [0.825-0.963] | 0.892 [0.826-0.964] | 0.898 [0.830-0.973] |
| Gross domestic product (milliard ISK) ^α^ | 1.012 [1.009-1.015] | 1.011 [1.004-1.019] | 1.011 [1.004-1.019] | 1.012 [1.004-1.020] |
| Balance of account (milliard ISK) ^β^ | 1.003 [1.001-1.005] | 0.997 [0.995-0.999] | 0.997 [0.995-0.999] | 0.997 [0.995-0.999] |
| Very difficult making ends meet (%)^β^ | 1.096 [1.054-1.140] | 0.881 [0.819-0.948] | 0.881 [0.819-0.948] | 0.878 [0.814-0.947] |
| Defaults on loans or rent (%)^β^ | 1.063 [1.032-1.095] | 0.917 [0.875-0.962] | 0.917 [0.875-0.962] | 0.915 [0.872-0.961] |

*Adjusted for time in weeks (time-trend).

** Adjusted for maternal age, gravidity and time in weeks [time-trend].

*** Simultaneously adjusted for maternal age, gravidity, time in weeks, gender, diabetes, pre-existing hypertension, relationship status, place of residence, employment status and citizenship.

^α^ On Quarterly basis

^β^ On yearly basis
