# Supplementary material for: Pregnancy-Induced Hypertensive Disorders before and after a National Economic Collapse: A Population Based Cohort Study
Source: PLoS One. 2015 Sep 17;10(9):e0138534. doi: 10.1371/journal.pone.0138534 (PMC4575018; doi:10.1371/journal.pone.0138534)
Supplement: S2 Appendix — The odds ratios [OR] and 95% confidence intervals [CI] of (A) overall pregnancy-induced hypertensive disorders, (B) gestational hypertension and (C) preeclampsia in each of the four post-collapse years following the economic collapse in Iceland compared with pre-collapse period, adjusted for seasonality. (DOCX) [file pone.0138534.s002.docx]

**S2 Appendix**. The odds ratios [OR] and 95% confidence intervals [CI] of (A) overall pregnancy-induced hypertensive disorders, (B) gestational hypertension and (C) preeclampsia in each of the four post-collapse years following the economic collapse in Iceland compared with pre-collapse period, adjusted for seasonality.

| **Regression models** | **Pre-collapse period^a^** | **Post-collapse year 1^b^** | **Post-collapse year 2^c^** | **Post-collapse year 3^d^** | **Post-collapse year 4^e^** |
| --- | --- | --- | --- | --- | --- |
|  | **OR [95% CI]** | **OR [95% CI]** | **OR [95% CI]** | **OR [95% CI]** | **OR [95% CI]** |
| ***(A) Pregnancy-induced hypertensive disorders*** |  |  |  |  |  |
| Crude | 1.00 [ref.] | 1.12 [0.98-1.27] | 1.01 [0.88-1.16] | 1.06 [0.92-1.22] | 1.04 [0.90-1.21] |
| Model I* | 1.00 [ref.] | 1.13 [0.94-1.36] | 1.03 [0.81-1.30] | 1.11 [0.83-1.47] | 1.09 [0.78-1.52] |
| Model II** | 1.00 [ref.] | 1.12 [0.93-1.36] | 1.02 [0.80-1.29] | 1.10 [0.82-1.47] | 1.06 [0.76-1.49] |
| Model III*** | 1.00 [ref.] | 1.11 [0.83-1.49] | 1.02 [0.74-1.40] | 1.10 [0.79-1.53] | 1.09 [0.77-1.53] |
| ***(B) Gestational hypertension*** |  |  |  |  |  |
| Crude | 1.00 [ref.] | 1.62 [1.37-1.93] | 1.29 [1.06-1.56] | 1.29 [1.06-1.57] | 1.28 [1.04-1.57] |
| Model I* | 1.00 [ref.] | 1.47 [1.13-1.91] | 1.11 [0.79-1.57] | 1.09 [0.72-1.65] | 1.04 [0.64-1.70] |
| Model II** | 1.00 [ref.] | 1.44 [1.10-1.87] | 1.08 [0.77-1.52] | 1.05 [0.69-1.59] | 1.01 [0.62-1.65] |
| Model III*** | 1.00 [ref.] | 1.12 [0.76-1.65] | 0.85 [0.55-1.32] | 0.90 [0.57-1.41] | 0.95 [0.59-1.56] |
| ***(C) Preeclampsia*** |  |  |  |  |  |
| Crude | 1.00 [ref.] | 0.78 [0.65-0.94] | 0.83 [0.69-1.00] | 0.89 [0.74-1.07] | 0.91 [0.75-1.11] |
| Model I* | 1.00 [ref.] | 0.86 [0.67-1.11] | 0.97 [0.71-1.31] | 1.09 [0.76-1.57] | 1.16 [0.76-1.77] |
| Model II** | 1.00 [ref.] | 0.85 [0.66-1.11] | 0.97 [0.71-1.32] | 1.11 [0.77-1.61] | 1.13 [0.74-1.75] |
| Model III*** | 1.00 [ref.] | 1.10 [0.73-1.64] | 1.24 [0.80-1.93] | 1.32 [0.85-2.06] | 1.29 [0.82-2.02] |

* Adjusted for maternal age, gravidity, time in weeks [time-trend] and seasonality.

** Simultaneously adjusted for maternal age, gravidity, time in weeks, seasonality, sex, diabetes, pre-existing hypertension, relationship status, place of residence, employment status and citizenship.

***Adjusted for maternal age, gravidity, time in weeks, seasonality and aggregate unemployment rate.

Included in the collapse groups are women with singleton pregnancies with gestational length of 20 weeks or more during ^a^September 27^th^ 2004 - September 28^th^ 2008, ^b^September 29^th^ 2008 – September 27^th^ 2009, ^c^September 28^th^ 2009 – October 3^rd^ 2010, ^d^October 4^th^ 2010 – October 2^nd^ 2011, ^e^October 3^rd^ 2011 – October 1^st^ 2012.
