# Supplementary material for: Pregnancy-Induced Hypertensive Disorders before and after a National Economic Collapse: A Population Based Cohort Study
Source: PLoS One. 2015 Sep 17;10(9):e0138534. doi: 10.1371/journal.pone.0138534 (PMC4575018; doi:10.1371/journal.pone.0138534)
Supplement: S4 Appendix — The odds ratios [OR] and 95% confidence intervals [CI] of (A) gestational hypertension and (B) β-blockers in the first and second half year following the economic collapse in Iceland compared with pre-collapse period (DOCX) [file pone.0138534.s004.docx]

**S4 Appendix.** The odds ratios [OR] and 95% confidence intervals [CI] of (A) gestational hypertension and (B) β-blockers in the first and second half year following the economic collapse in Iceland compared with pre-collapse period.

| **Regression models** | **Pre-collapse period^a^** | **4^th^ quarter 2008 &**  **1^st^ quarter 2009^b^** | **2^nd^ quarter 2009 &**  **3^rd^ quarter 2009^c^** |
| --- | --- | --- | --- |
|  | **OR [95% CI]** | **OR [95% CI]** | **OR [95% CI]** |
| ***(A) Gestational hypertension*** |  |  |  |
| Crude | 1.00 [ref.] | 1.52 [1.21-1.92] | 1.70 [1.36-2.12] |
| Model I* | 1.00 [ref.] | 1.36 [1.00-1.85] | 1.44 [1.05-1.97] |
| Model II** | 1.00 [ref.] | 1.34 [0.99-1.83] | 1.40 [1.02-1.93] |
| Model III*** | 1.00 [ref.] | 1.04 [0.66-1.67] | 1.16 [0.76-1.77] |
| ***(B) β-blockers*** |  |  |  |
| Crude | 1.00 [ref.] | 1.63 [1.26-2.10] | 1.66 [1.30-2.12] |
| Model I* | 1.00 [ref.] | 1.37 [0.98-1.92] | 1.31 [0.92-1.85] |
| Model II** | 1.00 [ref.] | 1.46 [1.03-2.06] | 1.37 [0.96-1.96] |
| Model III*** | 1.00 [ref.] | 1.13 [0.67-1.91] | 1.12 [0.70-1.80] |

* Adjusted for maternal age, gravidity, time in weeks [time-trend] and seasonality.

** Simultaneously adjusted for maternal age, gravidity, time in weeks, seasonality, sex, diabetes, pre-existing hypertension, relationship status, place of residence, employment status and citizenship.

***Adjusted for maternal age, gravidity, time in weeks, seasonality and aggregate unemployment rate.

Included in the collapse groups are women with singleton pregnancies with gestational length of 20 weeks or more during ^a^September 27^th^ 2004 - September 28^th^ 2008, ^b^September 29^th^ 2008 – March 29^th^ 2009, ^c^March 30^th^ 2009 – September 29^th^ 2009.
